# On Study of Immune Response to Tumor Cells in Prey-Predator System

**DOI:** 10.1155/2014/346597

**Published:** 2014-09-22

**Authors:** Gurpreet Kaur, Naseem Ahmad

**Affiliations:** ^1^CIRBSc., Jamia Millia Islamia, Jamia Nagar, New Delhi 110025, India; ^2^Department of Mathematics, Jamia Millia Islamia, Jamia Nagar, New Delhi 110025, India

## Abstract

This paper aims to develop the mathematical model that explores the immune response to a tumor system as a prey-predator system. A deterministic model defining the dynamics of tumor growth progression and regression has been analyzed. Our analysis indicates the tumor recurring and dormancy on the cellular level in combination with resting and hunting cells. The model considered in the present study is a generalization of El-Gohary (2008) by introducing the Michaelis-Menten function. This function describes the stimulation process of the resting cells by the tumor cells in the presence of tumor specific antigens. Local and global stability analysis have been performed along with the numerical simulation to support our findings.

## 1. Introduction

Cancer is one of the leading causes of death worldwide. Each year, the American Cancer Society projects the number of new cancer cases and death to estimate the contemporary cancer burden. In 2014, there will be an estimated 16 lakh new cancer cases diagnosed and around 5 lakh cancer deaths in the U.S. [1]. Not much is known about its mechanisms of establishment and destruction. It is the second fatal disease after the cardiovascular diseases [2]. According to World Economic Forum (WEF), cancer is among one of the three greatest risks to the global economy due to escalating cost of care, the threat to productivity from death and disability, and the effects of costs on household impoverishment [3].

The body is made up of many types of cells. Normally, cells grow and divide to produce new cells in a controlled and orderly manner. Sometimes, however, new cells continue to be produced when they are not needed. As a result, a mass of extra tissue called a tumor may develop. Tumor can be cancerous (malignant) or noncancerous (benign). A benign tumor is a known as tumor cell mass that does not fragment and spread beyond its original area of growth. Generally, benign impact on the body is not harmful and easy to be treated. Benign tumor can be harmful by growing large enough to interfere with normal body functions. Malignant tumors are nonencapsulated growths of tumor cells that are harmful; they have no wall or clear-cut border and may spread or invade other parts of the body normal tissue. In course of time, the cancer cells interfere with the normal functioning of organs via lymph or blood vessels [4, 5]. This stage is known as secondary tumor. The development of the cancerous cells is very complex and involves interaction of the various cells. In human anatomy, the immune system is triggered to quest and eradicate the tumor cells when they are detectable as nonself [6–9].

Researches are going in two directions to fight with cancer: one is experimental and the other is theoretical, that is, mathematical. Both experimentalists and theoreticians join hands to get rid of deadly disease, that is, cancer [10–13]. The studies done in [14] provide a comprehensive overview of the different approaches used in the modelling of the tumor-immune system interaction dynamics. A number of researches had been done so far which we referred to as Kuznetsov et al. [7], Saleem and Agrawal [15], De Pillis et al. [16, 17], Zhivkov and Waniewski [18], Galach [19], Babbs [20], and Ahmad and Kaur [21], by using ordinary differential equations, and tried to investigate the interaction among tumor cells and different type of immune cells. Kuznetsov et al. [7] study nonlinear dynamics of immunogenic tumors and manifest a number of phenomena as immunostimulation of tumor growth and sneaking through and formation of a dormant tumor. Galach [19] concluded in his simplified model that tumor was in dormant state, but in time delay model there was recurring of tumor in the presence of immune cells.

For understanding the interaction between tumor and immune cells, several researchers used the concept of prey-predator [2, 7, 15–17, 19, 22, 23]. There is a difference between the classical prey-predator model and tumor-immune system prey-predator model; it is that in the latter model survival of the immune population does not depend on the number of the preys (tumor cells). The immune cells play the role of the predator while tumor cells of prey. The cellular immune response identifies and eliminates the tumor cells from the host because tumor cells produce some antigens on its cell surface. The strength of the immune response depends on the tumor antigenicity [8, 24–26]. The cellular response is carried by T lymphocytes. These T helper cells cannot kill tumor cells, but they send urgent biochemical signals to a special type of effector cells called cytotoxic T lymphocytes. These effector cells eliminate cancerous cells by mounting a cytotoxic reaction that lyses their target [7, 19, 27].

Cancer self-remission model using stochastic approach is studied by Sarkar and Banerjee [22]. El-Gohary [23] extended the work of Sarkar and Banerjee [22] by providing an optimal control strategy that turned unstable steady states into asymptotically stable. The goal of the present paper is to modify the existing model of El-Gohary [23] by introducing a function called Michaelis-Menten. When the resting cells bind to tumor cells, they release cytokines, which act to increase its affinity. We introduce Michaelis-Menten function in this paper and we observe that the introduction of this particular function helps us to get stability as the growth rate of resting cells increases.

## 2. Mathematical Model

In this section, we considered tumor progression and regression as a prey-predator like system. The predator is the immune system which slaughters the tumor cells (prey). In most of the mathematical models of the tumor-immune system, the response of the immune system is considered as a single population of cells, namely, effector cell [2, 7, 16, 19], which perform the task of destroying cancer cells. This simplifying assumption allows decreasing the complexity of the dynamics of the immune system.

The predator, that is, immune system, is eradicating tumor cells in two stages: one is hunting cells and another is resting cells. Here, we are considering that hunting cells can slaughter tumor cells, but resting cells cannot. The cellular immune response identifies and eliminates the tumor cells from the host because tumor cells produce some antigens on its outer surface. The strength of the immune response depends on the tumor antigenicity [8, 24–26]. The cellular response of the immune system is carried by T lymphocytes. During maturation, T cells surface contains specialized antibody like receptors that see fragments of antigens on the surface of tumor cells. In most of the cases, T cells can recognize only antigen that is bound to a cell membrane protein called major histocompatibility complex (MHC) molecule. MHC molecule is a protein recognized by resting T cells, which distinguish between self and nonself. Resting T cells engulf the tumor cells and then produce various growth factors known collectively as cytokines, but they cannot kill tumor cells. Cytokines are chemical messenger switches which turn on the cytotoxic T lymphocytes (hunting cells). In contrast to the resting T cell, the cytotoxic T lymphocytes generally not only secrete many cytokines but also eliminate tumor cells by mounting a cytotoxic reaction that lyses their target [7, 19, 27].

Considering the above biological mechanism, we have produced a mathematical model of tumor development in immune response. The model involves certain assumptions as follows:

- logistic growth function is assumed for the growth of tumor cells in the absence of hunting CTL cells;
- the tumor cells and hunting cells are being eradicated at a rate proportional to the densities of tumor cells and hunting predator cells according to the law of mass action;
- the resting predator cells are converted to the hunting cells, either by direct contact with them or by contact with a fast diffusing substance (cytokines) produced by the hunting cells;
- resting cells also follow logistic growth in absence of tumor cells;
- once a hunting T cell has been converted, it will never return to the resting stage;
- resting cells also were stimulated due to the presence of tumor cells, and this is considered by the Michaelis-Menten function.

Let *T*(*t*) be the concentration of tumor cell in the given physiologic space at time *t*, *H*(*t*) the concentration of hunting T cells, and *R*(*t*) the concentration of resting T cells. The model governing the interaction between *T*(*t*), *H*(*t*), and *R*(*t*) is given by the following system of equations:

(1) dTdt=q+r1T(1−Tk1)−α1TH,dHdt=βHR−d1H−α2HT,dRdt=r2R(1−Rk2)−βHR−d2R+ρTRT+η,

where *q* is conversion rate of normal cells into tumor cells, *r*_1_ is growth rate of tumor cells, *k*_1_ is maximum carrying capacity of tumor cells, *α*_1_ is rate of killing of tumor cells by hunting cells, *β* is conversion rate of resting cells into hunting cells, *d*_1_ is apoptosis rate of hunting cells, *α*_2_ is rate of killing of hunting cells by tumor cells, *r*_2_ is growth rate of resting cells, *k*_2_ is maximum carrying capacity of resting cells, *d*_2_ is apoptosis rate of resting cells, *ρ* is proliferation rate of resting cells, and *η* is half-saturation for proliferation term.

Define the following dimensionless variables to reduce the number of the system parameters:

(2) $$\begin{array}{c} t^{*}=\frac{qt}{k_{1}},\;\;T^{*}=\frac{T}{k_{1}},\;\;H^{*}=\frac{\alpha_{1}k_{1}H}{q},\;\;R^{*}=\frac{R}{k_{2}}. \end{array}$$

Incorporating the dimension variables in the system of (1) and suppressing the star (∗) for our convenience, we get

(3) dTdt=1+a1T(1−T)−TH,

(4) dHdt=a2HR−a3H−a4HT,

(5) dRdt=a5R(1−R)−a6HR−a7R+a8TRT+K,

where

(6) a1=r1k1q,  a2=βk1k2q,  a3=k1d1q,a4=α2k12q,  a5=r2k1q,  a6=βα1,a7=k1d2q,  a8=k1ρq,  K=ηk1.

### 2.1. Determination of Equilibrium

To find the equilibrium point, we put the time rate of change as zero. Therefore, the system of (3)–(5) reduces to

(7) $$\begin{array}{c} 1+a_{1}T\left(1-T\right)-TH=0,\\ H=0,\;\;a_{2}R-a_{3}-a_{4}T=0,\\ R=0,\;\;a_{5}\left(1-R\right)-a_{6}H-a_{7}+\frac{a_{8}T}{T+K}=0. \end{array}$$

The solution of (7) in the *THR* space gives us the equilibrium points and coexistence equilibrium *E*_4_(*T*_4_, *H*_4_, *R*_4_). We observe the following.

(i) The equilibrium point lies on the boundary on the positive octant; that is,
  (8) $$\begin{array}{c} E_{1}=\left(\frac{1}{2}\left(1+\sqrt{1+\frac{4}{a_{1}}}\right),0,0\right). \end{array}$$
 This will always exist.
 (ii) The equilibrium point *E*_2_ lies in a *T*-*R* plane; that is,
  (9) $$\begin{array}{c} E_{2}\left(T_{2},0,R_{2}\right),\;\text{where}\;\;T_{2}=\frac{1}{2}\left(1+\sqrt{1+\frac{4}{a_{1}}}\right),\\ R_{2}=\frac{1}{a_{5}}\left(a_{5}-a_{7}+\frac{a_{8}T_{2}}{T_{2}+K}\right), \end{array}$$
 where *E*_2_ exists only when *a*_5_ + (*a*_8_ *T*_2_/(*T*_2_ + *K*)) > *a*_7_ for all positive parameters.
(iii) The equilibrium point *E*_3_ is in the *T*-*H* plane, that is,
  (10) $$\begin{array}{c} E_{3}\left(T_{3},H_{3},0\right),\;\text{where}\;\;T_{3}=-\frac{a_{3}}{a_{4}}. \end{array}$$
  In this case, we see that *T*_3_ is negative, so this point may not be taken into account.
(iv) Coexisting equilibrium point is *E*_4_(*T*_4_, *H*_4_, *R*_4_).
 Furthermore, eliminating *H* and *R* between the system of (7), we get a polynomial of degree three; that is,
  (11) $$\begin{array}{c} C_{3}T^{3}+C_{2}T^{2}+C_{1}T+C_{0}=0, \end{array}$$
 where
  (12) C3=a1a6−a4a5a2,C2=a5−a7+a8−a5a2(a3+a4K)−a1a6(1−K),C1=a5K−(a7K+a5a3Ka2+a6(a1K+1)),C0=−a6K<0.

The coefficients are not numbers here; they are functions of some model parameters like *a*_0_, *a*_1_, *a*_2_, and so forth, so the zero of this polynomial may not be obtained directly. Here, *C*_0_ is negative clearly while other coefficients are not defined in the sign, but the model parameters determine the sign of these coefficients.

### 2.2. Local Stability of the Prospective Equilibrium Points

The local asymptotic stability of each nonnegative equilibrium point has been studied by computing the variational matrix and finding the eigenvalues. For the stability of equilibrium points, the real parts of eigenvalues of variational matrix must be negative.

The variational matrix due to linearization of the system equations (3)–(5) at the arbitrary equilibrium point *E*_0_ (*T*, *H*, *R*) is given by

(13) $$\begin{array}{c} V_{0}=\left[\begin{array}{ccc} a_{1}-2a_{1}T-H & -T & 0\\ -a_{4}H & a_{2}R-a_{3}-a_{4}T & a_{2}H\\ \frac{a_{8}R}{T+K}-\frac{a_{8}RT}{{\left(T+K\right)}^{2}} & -a_{6}R & a_{5}-2a_{5}R-a_{6}H-a_{7}+\frac{a_{8}T}{T+K} \end{array}\right]. \end{array}$$

The local dynamical behavior of equilibrium points are investigated and obtained results by computing the variational matrices corresponding to each equilibrium point. The local asymptotic stability for each equilibrium point has been analyzed as follows

(i) The variational matrix at *E*_1_ is
  (14) $$\begin{array}{c} V_{1}=\left[\begin{array}{ccc} -\sqrt{1+\frac{4}{a_{1}}} & -\frac{1}{2}\left(1+\sqrt{1+\frac{4}{a_{1}}}\right) & 0\\ 0 & -a_{3}-\frac{a_{4}}{2}\left(1+\sqrt{1+\frac{4}{a_{1}}}\right) & 0\\ 0 & 0 & a_{5}-a_{7}+\frac{\left(a_{8}/2\right)\left(1+\sqrt{1+\left(4/a_{1}\right)}\right)}{\left(1/2\right)\left(1+\sqrt{1+\left(4/a_{1}\right)}\right)+K} \end{array}\right]. \end{array}$$

The eigenvalues of the *V*_1_ are $\lambda_{1}=-\sqrt{1+\left(4/a_{1}\right)}$ (<0), $\lambda_{2}=-\left(a3+\left(4/a_{1}\right)\left(1+\sqrt{1+\left(4/a_{1}\right)}\right)\right)$ (<0), and $\lambda_{3}=a_{5}-a_{7}+\left(\left(a_{8}/2\right)\left(1+\sqrt{1+\left(4/a_{1}\right)}\right)/(\left(1/2\right)\left(1+\sqrt{1+\left(4/a_{1}\right)}\right)+K)\right)$ (>0). Observing the sign of eigenvalues, we can state the following theorem.

**Theorem 1 .**

*E*_1_ is always a saddle point if *E*_2_ exists with locally unstable manifold along *R* direction and with the local stable manifold in *T*-*H* plane.

(ii) The variational matrix at *E*_2_ is

(15) V2=[a1−2a1T2−T200a2R2−a3−a4T20a8R2K(T2+K)2−a6R2a5−2a5R2−a7+a8T2T2+K].

The eigenvalues of this matrix are $\lambda_{1}=-\sqrt{1+\left(4/a_{1}\right)}$ (<0), *λ*_2_ = *a*_2_ *R*_2_ − *a*_3_ − *a*_4_ *T*_2_, and *λ*_3_ = −(*a*_5_ − *a*_7_ + (*a*_8_ *T*_2_/(*T*_2_ + *K*))) (<0 is the existence condition of *E*_2_). So, we state the following theorem.

**Theorem 2 .**

If *λ*_2_ < 0, then *E*_2_ is stable asymptotically in *T*-*R* plane, but if *λ*_2_ < 0 does not hold, then it will be saddle point with stable manifold in *T*-*R* plane and with unstable manifold in *H* direction.

(iii) The variational matrix at *E*_4_ is

(16) $$\begin{array}{c} V_{4}=\left[\begin{array}{ccc} a_{1}-2a_{1}T_{4}-H_{4} & -T_{4} & 0\\ -a_{4}H_{4} & a_{2}R_{4}-a_{3}-a_{4}T_{4} & a_{2}H_{4}\\ \frac{a_{8}R_{4}K}{{\left(T_{4}+K\right)}^{2}} & -a_{6}R_{4} & a_{5}-2a_{5}R_{4}-a_{6}H_{4}-a_{7}+\frac{a_{8}T_{4}}{T_{4}+K} \end{array}\right]. \end{array}$$

As *R*_4_ = (*a*_3_ + *a*_4_ *T*_4_)/*a*_2_, therefore, the eigenvalues of *V*_4_ are obtained as follows:

(17) $$\begin{array}{c} \text{Det}\left(V_{4}\right)=\left|\begin{array}{ccc} A-\lambda & -T_{4} & 0\\ -a_{4}H_{4} & -\lambda & a_{2}H_{4}\\ \frac{a_{8}R_{4}K}{{\left(T_{4}+K\right)}^{2}} & -a_{6}R_{4} & B-\lambda \end{array}\right|=0, \end{array}$$

where *A* = *a*_1_ − 2*a*_1_ *T*_4_ − *H*_4_ and *B* = *a*_5_ − 2*a*_5_ *R*_4_ − *a*_6_ *H*_4_ − *a*_7_ + (*a*_8_ *T*_4_/(*T*_4_ + *K*)). Consider

(18) Det(V4)=(A−λ)[(−λ)(B−λ)+a2a6R4H4] +T4[−a4H4(B−λ)−a2H4(a8R4K(T4+K)2)]⟹λ3−(A+B)λ2 +(AB+a2a6R4H4−a4H4T4)λ +(a4BH4T4+a2a8KH4T4R4(T4+K)2   −a2a6AH4R4a2a8KH4T4R4(T4+K)2)=0.

Define *A*_1_ = −(*A* + *B*), *A*_2_ = *AB* + *a*_2_ *a*_6_ *R*_4_ *H*_4_ − *a*_4_ *H*_4_ *T*_4_, and *A*_3_ = *a*_4_ *BH*_4_ *T*_4_  +  (*a*_2_ *a*_8_ *KH*_4_ *T*_4_ *R*_4_/(*T*_4_+*K*)^2^)  −  *a*_2_ *a*_6_ *AH*_4_ *R*_4_. Hence, we get *λ* ^3^  +  *A*_1_ *λ* ^2^  +  *A*_2_ *λ* + *A*_3_ = 0. Now, following Routh-Hurwitz criteria, the *λ*'s are negative if *A*_1_ > 0, *A*_3_ > 0, and *A*_1_ *A*_2_ − *A*_3_ > 0. We considered the following cases:

(a) *A*_1_ > 0⇒−(*A* + *B*) > 0.
 Which is only possible when *A* < 0 and *B* < 0. When
  (i) *A* < 0⇒*a*_1_ − 2*a*_1_ *T*_4_ − *H*_4_ < 0  that  is;  *a*_1_ < 2*a*_1_ *T*_4_ + *H*_4_;
  (ii) *B* < 0⇒*a*_5_ − 2*a*_5_ *R*_4_ − *a*_6_ *H*_4_ − *a*_7_ + (*a*_8_ *T*_4_/(*T*_4_ + *K*)) < 0  that  is;  *a*_5_ + (*a*_8_ *T*_4_/(*T*_4_ + *K*)) < 2*a*_5_ *R*_4_ + *a*_6_ *H*_4_ + *a*_7_;
(b) *A*_3_ > 0⇒*a*_4_ *BH*_4_ *T*_4_ + (*a*_2_ *a*_8_ *KH*_4_ *T*_4_ *R*_4_/(*T*_4_+*K*)^2^) − *a*_2_ *a*_6_ *AH*_4_ *R*_4_ > 0.
 As *A* < 0 and *B* < 0. Therefore, *A*_3_ > 0 for *a*_4_ *BH*_4_ *T*_4_ + (*a*_2_ *a*_8_ *KH*_4_ *T*_4_ *R*_4_/(*T*_4_+*K*)^2^) > *a*_2_ *a*_6_ *AH*_4_ *R*_4_;
(c) *A*_1_ *A*_2_ − *A*_3_ > 0⇒−(*A* + *B*)(*AB* + *a*_2_ *a*_6_ *R*_4_ *H*_4_ − *a*_4_ *H*_4_ *T*_4_)  −  (*a*_4_ *BH*_4_ *T*_4_ + (*a*_2_ *a*_8_ *KH*_4_ *T*_4_ *R*_4_/(*T*_4_+*K*)^2^) − *a*_2_ *a*_6_ *AH*_4_ *R*_4_)  >  0⇒*a*_4_ *AH*_4_ *T*_4_ > *A* ^2^ *B* + *AB* ^2^ + *a*_2_ *a*_6_ *BR*_4_ *H*_4_ + (*a*_2_ *a*_8_ *KH*_4_ *R*_4_ *T*_4_/(*T*_4_+*K*)^2^). Hence we arrive at a conclusion.

**Theorem 3 .**

If *A*_1_ > 0, *A*_3_ > 0, and *A*_1_ *A*_2_ − *A*_3_ > 0, then *E*_4_ is locally asymptotically stable in *T*-*H*-*R* plane.

### 2.3. Global Stability of the System

The system considered in this paper is determined by three nonnegative equilibrium points *E*_1_, *E*_2_, and *E*_4_. Out of these points, *E*_1_ is unstable; therefore, our aim is to verify the global stability of the system through *E*_2_ and *E*_4_. Hence, we check the global stability of these points as follows.

**Theorem 4 .**

If the equilibrium point *E*_2_ is locally asymptotically stable in the interior of a positive quadrant of *T*-*R* plane, then it will be globally asymptotically stable there.

**Proof**

Define Dulac function *H*_1_(*T*, *R*) = (1/*TR*); *T*, *R* > 0. Using the system of (3) and (5), let

(19) f(T,R)=1+a1T(1−T),g(T,R)=a5R(1−R)−a7R+a8TR(T+K).

Now,

(20) Div(H1f,H1g)=∂∂T(fH1)+∂∂R(gH1)=−(1RT2+a1R+a5T)<0.

It is observed here that Div(*H*_1_ *f*, *H*_1_ *g*) does not change its sign in the positive quadrant of *T*-*R* plane. By Bendixson-Dulac criterion, there is no limit cycle or homoclinic connection observed in the positive quadrant of the *T*-*R* plane. Hence, if *E*_2_ is locally asymptotically stable, then it will be globally asymptotically stable in the interior of a positive quadrant of *T*-*R* plane.

**Theorem 5 .**

If the equilibrium point *E*_4_ is locally asymptotically stable in the interior of the positive octant of *THR* space then it will be globally asymptotically stable there.

**Proof**

Consider the following Lyapunov function around *E*_4_(*T*_4_, *H*_4_, *R*_4_):

(21) V=(T−T4−T4ln⁡TT4)+(H−H4−H4ln⁡HH4) +(R−R4−R4ln⁡RR4).

Differentiating *V* with respect to *t*, we get

(22) $$\begin{array}{c} \overset{•}{V}=\left(T-T_{4}\right)\frac{\overset{•}{T}}{T}+\left(H-H_{4}\right)\frac{\overset{•}{H}}{H}+\left(R-R_{4}\right)\frac{\overset{•}{R}}{R}. \end{array}$$

Using (3)–(5) in $\overset{•}{V}$, we have

(23) V•(T,H,R)  =−[(a1+1TT4)(T−T4)2+a5(R−R4)2iiiiiiiiiiiiiii+(1+a4)(T−T4)(H−H4)iiiiiiiiiiiiiii+(a6−a2)(R−R4)(H−H4)(a1+1TT4)iiiiiiiiiiiiiii−a8K(T+K)(T4+K)(R−R4)(T−T4)].

Thus, $\overset{•}{V}=\left(T,H,R\right)$ is a quadratic form which can be expressed as $\overset{•}{V}=-X^{T}AX$, where *X* ^*T*^ = (*T* − *T*_4_, *H* − *H*_4_, *R* − *R*_4_) and *A* is a symmetric matrix given by

(24) $$\begin{array}{c} A=\left(\begin{array}{ccc} a_{11} & a_{12} & a_{13}\\ a_{21} & a_{22} & a_{23}\\ a_{31} & a_{32} & a_{33} \end{array}\right), \end{array}$$

with *a*_11_ = (*a*_1_ + (1/*TT*_4_)),  *a*_12_ = (1 + *a*_4_)/2,  *a*_13_ = −(*a*_8_ *K*/2(*T* + *K*)(*T*_4_ + *K*)), *a*_22_ = 0, *a*_23_ = (*a*_6_ − *a*_2_)/2, and *a*_33_ = *a*_5_.

$\overset{•}{V}$ is negative definite if *a*_11_ > 0, *a*_33_ > 0, and *a*_13_^2^ − *a*_11_ *a*_33_ ≤ 0. Hence, the *V* is a Lyapunov function with respect to *E*_4_.

## 3. Simulation and Discussion

**Figure 1:**
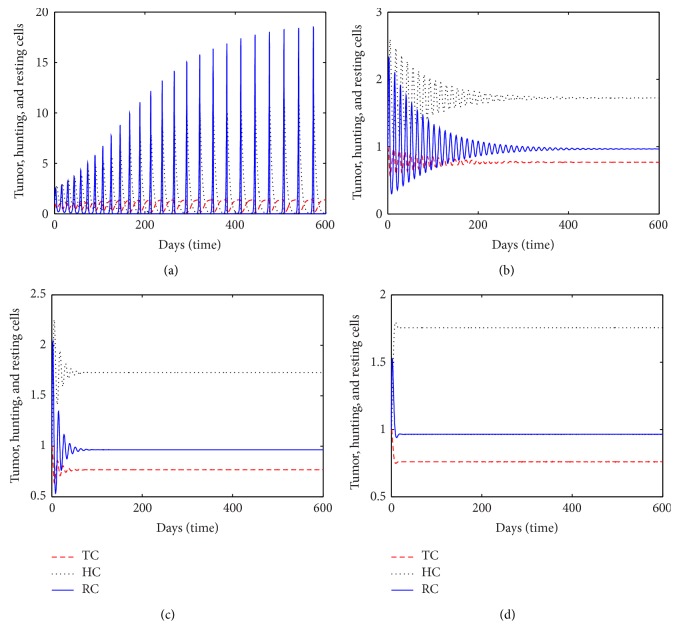
Tumor cells, hunting cells, and resting cells with time for *a*_1_ = 1.82, *a*_2_ = 0.239, *a*_3_ = 0.2, *a*_4_ = 0.04, *a*_6_ = 0.5, *a*_7_ = 0.01, *a*_8_ = 2, *k* = 1, (a) *a*_5_ = 0.0191, (b) *a*_5_ = 0.0691, (c), *a*_5_ = 0.191, and (d) *a*_5_ = 0.6291.

**Figure 2:**
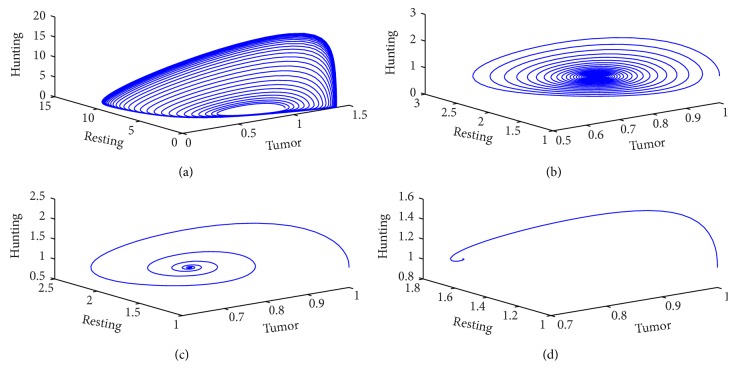
Phase portraits corresponding to the system (3)–(5) for the following parameter values: *a*_1_ = 1.82, *a*_2_ = 0.239, *a*_3_ = 0.2, *a*_4_ = 0.04, *a*_6_ = 0.5, *a*_7_ = 0.01, *a*_8_ = 2, *k* = 1, (a) *a*_5_ = 0.0191, (b) *a*_5_ = 0.0691, (c) *a*_5_ = 0.191, and (d) *a*_5_ = 0.6291.

This section is devoted to the study of a mathematical model presented by the system of (3)–(5). Using Matlab solver and assuming the values of some parameters randomly, we try to reach the conclusions. Figures 1 and 2 have been obtained for different values of *a*_5_ by taking all others a's fixed to study the behavior of the model.

From a numerical simulation, we discovered that by introducing the simulating function in resting cell population due to the presence of tumor; while increase the growth rate of resting cells can control the progression of tumor. In Figure 1, we compare the temporal growth of the number of tumor cells, hunting cells, and resting cells for different values of the parameter *a*_5_. The level of interaction is maximum according to Figure 1(a) when *a*_5_ = 0.0191 which shows a sustained oscillation for different populations. Observing Figure 1(a) and its phase portrait Figure 2(a), we exhibit a stage of  “recurring” tumor [19, 28, 29]. The cyclic fluctuation reduces in different populations while increasing the value of *a*_5_. The sustains oscillation give a way to a stable spiral with quick damping, which leads to the persistent tumor, that could be described as dormant (A small stable tumor does not change in size is referred as dormant tumor). Leading towards the persistent tumor, that could be described as dormant (a small stable tumor which does not change in size is referred to as dormant tumor [19, 29]). This state is desirable clinical condition since the tumor growth is blocked. The phase portrait Figure 2(a) exhibits a trend that the recovery phase is weaker than the tumor phase. It has also been noticed that the sustained oscillation phase tends to damped oscillation phase for *a*_5_ = 0.046039. According to the damped phase in Figure 1(b), we see that hunting cells attack tumor cells together with resting cells for a short span of time and later hunting cells do not interfere either with resting cells or with tumor cells. From the rest of Figures 1(c), 1(d) and 2(b) to 2(d), it has been observed that, on increasing *a*_5_, the growth rate of resting cells, the tumor phase tends to recovery phase. Also, it has been seen that the system has not been reached to complete recovery phase; that is, tumor is not eliminated.

## 4. Conclusion

An interaction between tumor cells as prey and immune system which consists of resting and hunting cells as predator has been studied. We analyze the model with regard to local and global stability of equilibrium points. Global stability of steady state *E*_2_ is established using Bendixson-Dulac criteria and coexisting steady state *E*_4_ is done by Lyapunov function. Using the Michealis-Menten function approach, it has been observed that as *a*_5_, the growth of resting cells, increases, the behavior of tumor growth persists from recurring state to a dormant state. A better understanding of the influence of the immune system on dormancy could lead to the development of immunological therapies in order to prevent the progression of the tumour and then the switch from the state of dormancy to tumour growth. Our model may lead to novel detection techniques in the clinic and therapeutic options to prevent deadly tumor recurrence and metastasis.
